# Virtual reality perimetry compared to standard automated perimetry in adults with glaucoma: A systematic review

**DOI:** 10.1371/journal.pone.0318074

**Published:** 2025-01-24

**Authors:** Natan Hekmatjah, Chimelie Chibututu, Ying Han, Jeremy D. Keenan, Julius T. Oatts

**Affiliations:** 1 School of Medicine, University of California, San Francisco, San Francisco, California, United States of America; 2 University of Michigan Medical School, Ann Arbor, Michigan, United States of America; 3 Francis I. Proctor Foundation, San Francisco, San Francisco, California, United States of America; 4 Department of Ophthalmology, University of California, San Francisco, San Francisco, California, United States of America; 5 Division of Ophthalmology, Children’s Hospital of Philadelphia, Philadelphia, Pennsylvania, United States of America; The University of Iowa, UNITED STATES OF AMERICA

## Abstract

**Purpose:**

The purpose of this systematic review was to consolidate and summarize available data comparing virtual reality perimetry (VRP) with standard automated perimetry (SAP) in adults with glaucoma. Understanding the utility and diagnostic performance of emerging VRP technology may expand access to visual field testing but requires evidence-based validation.

**Methods:**

A systematic literature search was conducted in 3 databases (PubMed Central, Embase, and Cochrane Central Register of Controlled Trials) from the date of inception to 10/09/2024. Eligibility criteria included randomized controlled trials or prospective or retrospective cohort studies that compared different modalities of VRP to SAP in adults >18 years of age with glaucoma. Studies were excluded if they were review articles, letters, case reports, abstract-only papers, unavailable full text, or non-English language. Identified studies were formally evaluated for risk of bias using the Newcastle-Ottawa tool. The study protocol was prospectively registered with PROSPERO in May 2023 (registration number: CRD42023429071).

**Results:**

The literature search yielded 1657 results. After deduplication, abstract and title screening, 14 studies met inclusion criteria and were included in the final systematic review. Compared to Humphrey Field Analyzer or Octopus 900, 10 different VRP devices were included in our study: Oculus Quest, Smartphone-based Campimetry, Toronto Portable Perimeter, VirtualEye, Advance Vision Analyzer, VisuALL, Vivid Vision Perimeter, C3 fields visual field analyzer, Radius, and Virtual Field. Overall, published studies of VRP are promising; however, more work is required to better evaluate these devices, namely test-retest repeatability.

**Conclusions:**

VRP holds strong potential to evaluate visual fields in adults with glaucoma, though further data is needed to validate emerging technologies and testing protocols. Eye providers may consider using these devices to monitor certain adults with glaucoma.

## Introduction

Glaucoma is one of the leading causes of blindness and affects 80 million people worldwide [1]. Standard automated perimetry (SAP) is generally considered the clinical standard test to diagnose and monitor glaucomatous visual field loss. SAP can be performed on several devices including the Humphrey Field Analyzer (HFA, Carl Zeiss Meditec) and Octopus Perimeter (Haag-Streit). SAP has several limitations: it requires monocular occlusion, strict patient positioning, and maintained central fixation, all of which can make the test time-consuming and tiring [2]. Tests are typically performed 1–2 times per year and inter-test variability can reduce the accuracy and clinical utility of measurements. Newer technologies are emerging with the goal of providing accessible and innovative ways of assessing and monitoring visual field loss in adults with glaucoma [2].

Virtual reality perimetry (VRP), using virtual reality headsets to create an immersive environment in which visual field testing is performed, has gained increasing attention due to its portability and high levels of acceptability among patients [3]. For these reasons, early iterations of VRP have proven to be a suitable alternative for visual field testing in patients unable to test on a perimeter, such as hospitalized patients [4]. Unlike SAP, which requires in-person clinic visits, VRP offers the advantage of being accessible anytime and anywhere. While newer VRP testing modalities have also been shown to detect visual field defects and demonstrate some correlation to the clinical standard perimetry, there has been no systematic review evaluating the evidence for VRP as a diagnostic tool in adults with glaucoma. This systematic review aims to consolidate and summarize available data on VRP and its diagnostic accuracy compared to SAP in adult glaucoma patients.

## Methods

### Eligibility criteria for considering studies for this review

Eligibility criteria for studies under consideration for this review included randomized controlled trials, prospective cohort studies, or retrospective cohort studies comparing different modalities of VRP to SAP in adults (> 18 years of age) with glaucoma. Non-randomized studies were included given the lack of robust randomized studies evaluating VRP for adults with glaucoma. Exclusion criteria included: review articles, letters, case reports, abstract-only papers, unavailable full text, non-English language papers, other unrelated articles, or studies comparing SAP to perimeters other than VRP. Outcomes of interest were the primary quantitative comparisons between SAP and VRP data reported in the included studies.

### Search methods for identifying studies

An a priori protocol with a predefined search strategy was used to search 3 databases: PubMed Central, Embase, and Cochrane Central Register of Controlled Trials (**S1 Protocol**). PubMed Central was searched first, followed by Embase, and then Cochrane Central Register of Controlled Trials. Studies were restricted to the English language and the search was conducted on 10/09/2024. For Cochrane Central Register of Controlled Trials, “All Text” was selected for each search. The exact full search strategy used for PubMed Central, Embase, and Cochrane Central Register of Controlled Trials with sufficient detail to permit replication is listed in the **S1 Appendix**. The 3 databases were searched by two independent reviewers (N.H. and C.C.) to ensure the same number of search results.

### Study selection

Title and abstract screening and study selection were performed and identified by hand by two independent reviewers (J.O. and C.C.), and any uncertainty was resolved by discussion and adjudication with a third party (N.H.).

### Data collection and risk of bias assessment

Data were extracted from eligible studies by a single reviewer (N.H.). The data extracted from included studies were: author names, year of publication, country, study design type, number of participants, mean age of study population, VRP device type, VRP system specifications, mean test duration, eye tracking status, standard perimeter device type, and primary outcome measures. Identified studies were formally evaluated for risk of bias assessment by two independent reviewers (N.H. and C.C.) using the Newcastle-Ottawa tool adapted for cross-sectional studies [5]. Studies with disagreement in the assessment of risk of bias between the two reviewers were resolved through discussion with a third reviewer (J.O.). The risk of bias domains assessed included Selection: representativeness of the cases, sample size, non-response rate, and ascertainment of the screening/surveillance tool; Comparability: potential confounders investigated by subgroup or multivariable analysis; and Outcome: assessment of the outcome, and statistical test. The maximum number of stars for each domain was 5, 1, and 3 for Selection, Comparability, and Outcome, respectively. As described previously, total star scores from 0–4, 5–6, and 7–9 were considered as having high, moderate, and low risk of bias, respectively [6].

### Data synthesis and analysis

The study protocol was prospectively registered with PROSPERO in May 2023 (registration number: CRD42023429071, accessed at https://www.crd.york.ac.uk/PROSPERO/) and was guided by the standards of the Preferred Reporting Items for Systematic Review and Meta-Analysis (PRISMA) Statement (**S1 Checklist**). No human subjects are included in this study, and thus exempt from institutional review board approval. The study adhered to the tenets of the Declaration of Helsinki.

## Results

**Fig 1:**
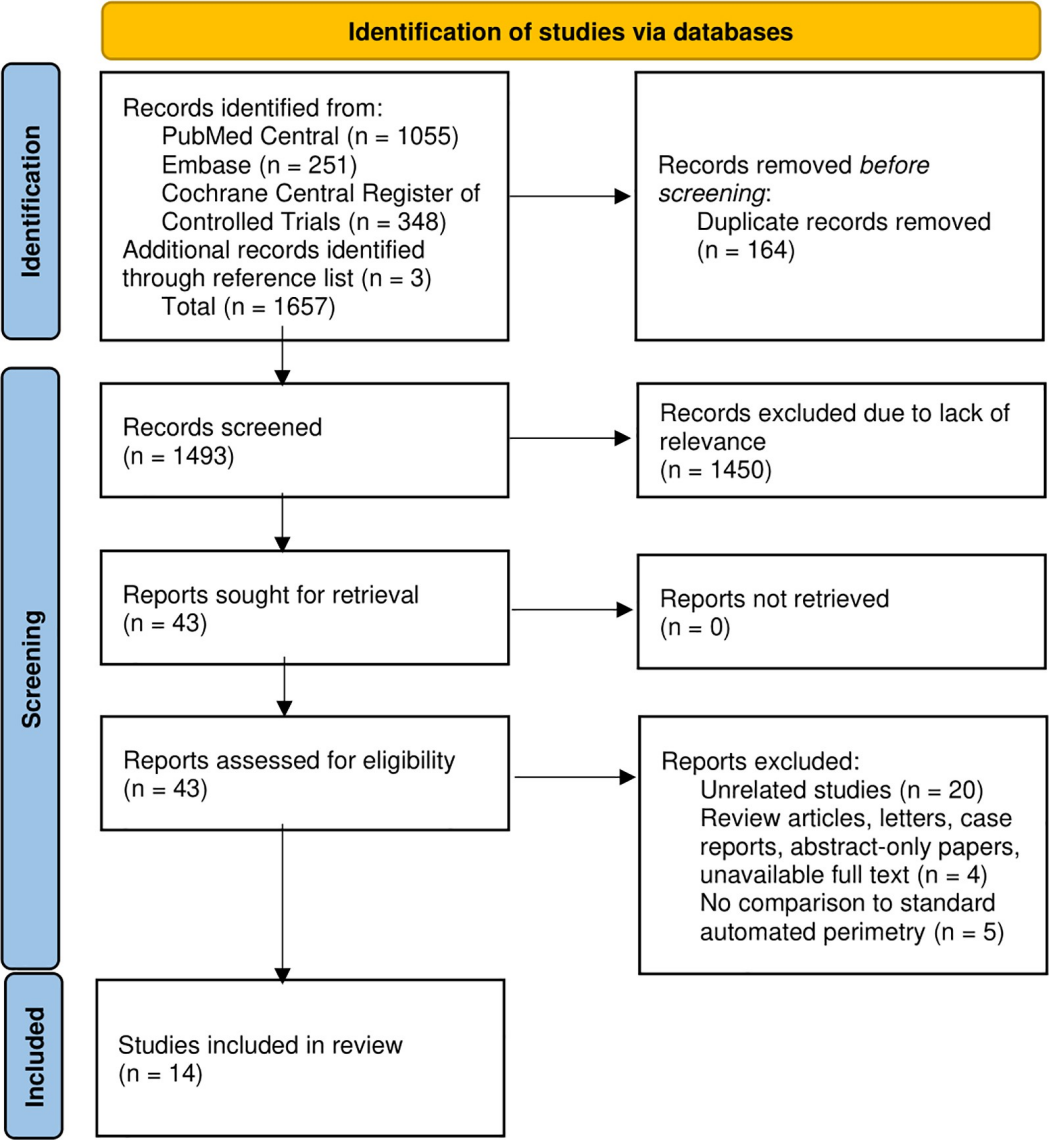
Flow diagram describing the process of study selection.

A total of 1657 articles were initially identified. After removal of duplicates (n = 164), 1493 articles underwent title and abstract screening by two authors (J.O. and C.C.), with 43 articles remaining for full-text retrieval for review. Of these, articles were excluded due to unrelated content (n = 20), review articles, letters, case reports, abstract-only papers, unavailable full text (n = 4), and no comparison to SAP (n = 5). Ultimately, 14 studies met the inclusion criteria and were included in the systematic review (**Fig 1**). Characteristics of the included studies are described in **Table 1**. With the exception of the Advanced Vision Analyzer, VisuALL, and Vivid Vision Perimeter, there was only one study per device and only around half of the studies included formal Bland-Altman analysis to assess agreement between VRP and SAP.

**Table 1:** Study characteristics.

Author (Year)	Country	Study Design	Subjects	Participant age (±SD, years)	VRP System	VR System Specifications	Test Duration per eye (minutes)	Eye tracking	SAP Device	Outcome Measures Comparing VRP and SAP; Bland-Altman Analysis
Stapelfeldt et al. (2021) [1]	Switzerland	Prospective, single-center	70 eyes of 70 subjects: 36 healthy, 34 glaucoma	_All subjects:_ 62.80±9.38 _Healthy subjects:_ 60.67±10.64 _Glaucoma subjects:_ 65.06±7.15	Oculus Quest virtual reality headset	Background luminance: 10 cd/m^2^ Stimulus: Goldmann size III light stimulus Threshold: Not described in depth	_All subjects:_ VRP median: 6.57 (6.4, 6.7) SAP median: 5.75 (5.7, 6.0) p<0.0001 _Healthy subjects:_ VRP median: 6.70 (6.5, 6.9) SAP median: 5.46 (5.4, 5.7) p<0.001 _Glaucoma subjects:_ VRP median: 6.39 (6.2, 6.7) SAP median: 6.27 (6.0, 6.7) p>0.1	No	Octopus 900 dynamic strategy	Difference in MD (±SD, dB): _All subjects:_ 0.6±2.3 _Healthy subjects:_ -0.1±2.2 _Glaucoma subjects:_ 1.4±2.1 Spearman correlation coefficients for MD: _All subjects:_ 0.77, p<0.00001 _Healthy subjects:_ 0.50, p<0.001 _Glaucoma subjects:_ 0.70, p<0.0001 No Bland-Altman analysis
Grau et al. (2023) [7]	Germany	Prospective, single-center	93 eyes of 93 subjects: 19 control, 11 pre-perimetric glaucoma, 46 perimetric glaucoma, 17 ocular hypertension	_Control subjects:_ 59.21±12.46 _Pre-perimetric glaucoma subjects:_ 64.36±11.74 _Perimetric glaucoma subjects:_ 64.72±12.27 _Ocular hypertension subjects:_ 59.06±11.22	Smartphone-based campimetry	Background luminance: 0.05 cd/m^2^ Stimulus: Goldmann size III stimulus Threshold: Full threshold staircase strategy using a standard 4/2 dB	Not reported	No	Octopus 900 normal and dynamic mode	Difference in MS (±SD, dB): 1.88±2.30, Bland-Altman 95% LOA (-2.63, 6.39) Spearman correlation coefficient for MS: r = 0.815, p<0.05 Spearman correlation coefficient for test-retest reliability: r = 0.591, p<0.05
Ahmed et al. (2022) [8]	Canada	Prospective, multicenter	150 eyes of 91 subjects: 114 mild glaucoma, 26 moderate glaucoma, 10 advanced glaucoma	_All subjects:_ 69.0±11.2	Toronto Portable Perimeter	Background luminance: 10 cd/m^2^ Stimulus: Goldmann size III, IV, and V stimuli Threshold: Zippy Estimation of Sequential Threshold (ZEST)	_All subjects:_ VRP: 5.78±0.78 SAP: 5.78±1.01 p = 0.92	No	Humphrey Field Analyzer 24–2 SITA Standard	Difference in MD (±SD, dB): 0.21±2.27, p = 0.74, R^2^ = 0.83 Bland-Altman 95% LOA (-4.25, 4.67) Difference in PSD (dB): -0.13±1.83, p = 0.71 R^2^ = 0.65 Bland-Altman 95% LOA (-3.72, 3.47) Difference in VFI (%): 0.66±5.90, p = 0.72, R^2^ = 0.87 Bland-Altman 95% LOA (-10.9, 12.3) Difference in test duration (seconds): 0.65±49.95, p = 0.92, R^2^ = 0.36 Bland-Altman 95% LOA (-97.5, 98.8)
Wroblewski et al. (2014) [10]	United States	Prospective, single center	62 subjects (eyes not specified): 17 control, 30 glaucoma, 6 glaucoma suspect, 9 “other”	_Virtual grasp (VG) versus HFA group (40 eyes):_ 63±13 _Manual (MAN) versus HFA group (59 eyes):_ 63±13	VirtualEye, Manual (MAN) and virtual grasp (VG) protocols	Background luminance: Not provided Stimulus: Goldmann size III stimulus Threshold: Full threshold 4/2 strategy	_MAN:_ 10.6±3.3 _VG:_ 9.4±2.1 _SAP:_ 6.1±1.0	Yes	Humphrey Field Analyzer 24–2 SITA Standard, 30–2 SITA FAST	Difference in MD (±SD, dB): _VG Group:_ -8.8±9.1 _MAN Group:_ -9.2±8.8 No p-values given, no Bland-Altman analysis
Narang et al. (2021) [11]	India	Prospective, single center	160 eyes of 160 subjects: 85 control, 75 glaucoma	_Control subjects:_ 38.21±15.59 _Glaucoma subjects:_ 56.72±13.15	Advanced Vision Analyzer	Background luminance: 9.6 cd/m^2^ Stimulus: Goldmann size III stimulus Threshold: Full Threshold, Elisar Standard, and Elisar Fast which use a 4/2 dB staircase procedure	_All subjects:_ VRP: 7.08±1.55 SAP: 6.26±0.54 p = 0.228	Yes	Humphrey Field Analyzer 24–2 SITA Standard	MD: _Control group:_ ICC = 0.177 Bland-Altman 95% LOA (-5.15, 4.47) _Glaucoma group:_ ICC = 0.93 Bland-Altman 95% LOA (-6.98, 6.76) PSD: _Control group:_ ICC = 0.367 Bland-Altman 95% LOA (-2.59, 1.29) _Glaucoma group:_ ICC = 0.738 Bland-Altman 95% LOA (-3.75, 4.33)
Narang et al. (2022) [13]	India	Prospective, single center	112 eyes of 112 subjects: 36 control, 66 glaucoma, 10 glaucoma suspect	_Control subjects:_ 41.69±15.86 _Glaucoma subjects:_ 61.08±14.46 _Glaucoma suspect subjects:_ 51.4±11.22	Advanced Vision Analyzer	Background luminance: 9.6 cd/m^2^ Stimulus: Goldmann size III stimulus Threshold: Full Threshold, Elisar Standard, and Elisar Fast which use a 4/2 dB staircase procedure	_All subjects:_ VRP: 7.14±1.46 SAP: 6.28±1.26 p = 0.0006	Yes	Humphrey Field Analyzer 10–2 SITA Standard	MD: _Control group:_ ICC = 0.51 Bland-Altman 95% LOA (-3.63, 3.82) _Glaucoma group:_ ICC = 0.96 Bland-Altman 95% LOA (-3.87, 3.89) _Glaucoma suspect group:_ ICC = 0.24 Bland-Altman 95% LOA (-4.08, 6.93)
Razeghinejad et al. (2021) [14]	United States	Prospective, single center, Cross-sectional	102 eyes of 51 subjects: 25 control, 26 mild and moderate glaucoma	_Control subjects:_ 53.96 (range, 30–79) _Glaucoma subjects:_ 66.04 (range, 23–86)	Olleyes VisuALL	Background luminance: 3 cd/m^2^ Stimulus: Goldmann size III stimulus Threshold: Full threshold testing algorithm	_Control subjects:_ VRP: 6.13 SAP: 4.77 p = 0.02 _Glaucoma subjects:_ VRP: 9.28 SAP: 5.62 p<0.001	Yes	Humphrey Field Analyzer 24–2 SITA Standard	Difference in MS: _Control group_ 0.25 Bland-Altman 95% LOA (-2.29, 2.80) _Glaucoma group_ 0.25 Bland-Altman 95% LOA (-4.69, 5.21) Spearman correlation coefficient for MS: _Control group_ 0.5, p = 0.001 _Glaucoma group_ 0.8, p<0.001
Berneshawi et al. (2024) [16]	United States	Prospective, single-center	16 eyes of 9 glaucoma subjects	_All subjects:_ 60.2±16.4	Olleyes VisuALL	Background luminance: 1 cd/m^2^ Stimulus: Goldmann size III stimulus Threshold: Proprietary threshold strategy	Exact duration not reported. However, VRP was found to be significantly shorter than SAP (p < 0.001)	No	Humphrey Field Analyzer 24–2 SITA Standard	Spearman correlation coefficient for MD: r = 0.8793, p < 0.001 ICC = 0.947 Spearman correlation coefficient for MS: r = 0.385–0.887 (variance across Garway-Health sectors; 5 of 6 p≤0.01) Bland-Altman 95% LOA (-3.98, 7.35)
Griffin et al. (2024) [15]	United States	Prospective, single-center	43 eyes of 24 glaucoma subjects	_All subjects:_ 69.1±16.5	Olleyes VisuALL	Background luminance: 1 cd/m^2^ Stimulus: Goldmann size III stimulus Threshold: Not reported	Not reported	No	Humphrey Field Analyzer 24–2 SITA Standard	Spearman correlation coefficient for MD: r = 0.871, p<0.001 Median difference in sensitivity (by locus): -0.3±1.5 dB Bland-Altman Performed but LOA not reported
Bradley et al. (2024) [17]	United States	Prospective, multicenter	100 eyes (62 OD, 38 OS) of 100 subjects; 50 suspect or mild glaucoma, 50 moderate or severe glaucoma	_All subjects:_ 69±11.1	Radius virtual reality perimeter	Background luminance: 10 cd/m^2^ Stimulus: Goldman III stimulus Threshold: Proprietary threshold strategy	_All subjects:_ VRP: 4.97 SAP: 5.68	No	Humphrey Field Analyzer 24–2 SITA Standard	Pearson correlation coefficient for MD: r = 0.94 (no p-value) Estimated sensitivities: OD: no differences (p = 0.017) OS: significant difference (p < 0.001) Test-retest: no differences (p > 0.019) Low agreement between 15 to 22 dB MD: r = 0.94 Bland-Altman analysis not performed
Phu et al. (2024) [18]	Australia	Prospective, single-center, Cross-sectional	95 eyes of 95 subjects: 41 control, 54 glaucoma	_Control subjects:_ 64.8±10.4 _Glaucoma subjects:_ 63.7±9.5	Virtual Field	Background luminance: 0.218 cd/m^2^ Stimulus: Goldmann size III stimulus Threshold: Fast Full Threshold algorithm	Exact duration nNot reported. However, SAP was was found, on average, to take 76 seconds longer than VRP (p < 0.0001)	No	Humphrey Field Analyzer 24–2 SITA Standard	Pearson correlation coefficient for MD: r = 0.87665, p< 0.0001 Bland-Altman 95% LOA (-3.48, 4.20), p = 0.91 ICC = 0.86 Pearson correlation coefficient for PSD: r = 0.9400, p < 0.0001 Bland-Altman 95% LOA (-3.19,3.41), p < 0.0001 ICC = 0.82 Pointwise sensitivity: r = 0.7765, p < 0.0001 Bland-Altman 95% LOA (-1.1, 13.4), p < 0.0001 ICC = 0.47 Fixation losses: VRP = 0.05, SAP = 0.13, p = 0.0006 False positive rates: VRP = 0.01, SAP = 0.02, p < 0.0001
Greenfield et al. (2021) [19]	United States	Prospective, single center, Cross-sectional	24 eyes of 12 subjects: 7 glaucoma, 5 glaucoma suspect	_Glaucoma subjects:_ 64.6±11.4 _Glaucoma suspect subjects:_ 61.8±6.5	Vivid Vision Perimeter (Swift protocol)	Background luminance: 25 cd/m^2^ Stimulus: Goldmann size III stimulus Threshold: Suprathreshold test	_All subjects:_ VRP: 8.5 SAP: 12.2 p<0.001	No	Humphrey Field Analyzer 24–2 SITA Standard	Pearson correlation coefficient for MS: 0.86, p<0.001 Bland-Altman analysis not performed comparing testing modalities
Chia et al. (2023) [20]	United States	Prospective, single center, Cross-sectional	36 eyes of 21 glaucoma subjects	_Glaucoma subjects:_ 62.2±10.8	Vivid Vision Perimeter (10 protocol)	Background luminance: 25 cd/m^2^ Stimulus: Goldmann size III stimulus Threshold: Suprathreshold test	_All subjects:_ VRP: 10.8±1.3 SAP: 5.9±0.8	No	Humphrey Field Analyzer 24–2 SITA Standard	Spearman correlation coefficient for MS (moderate and severe glaucoma [95%CI]): 0.87 (0.66, 0.98), p<0.001 Spearman correlation coefficient for MS (all eyes [95%CI]): 0.67 (0.28, 0.94), p<0.05 Bland-Altman analysis not performed
Mees et al. (2020) [21]	India	Prospective, single center	157 subjects (eyes not specified): 95 control, 62 glaucoma	_Control subjects:_ 49.8±9.2 _Glaucoma subjects:_ 54.2±9.3	C3 fields visual field analyzer	Background luminance: 4 cd/m^2^ Stimulus: 0.55 mm stimulus with 60 cd/m^2^ luminance Threshold: Suprathreshold test	_All subjects:_ VRP: 3.29 SAP: Not reported	No	Humphrey Field Analyzer 24–2 SITA Standard	Correlation coefficient (missed CFA stimuli versus HFA MD): 0.62, p<0.001 Correlation coefficient (missed CFA stimuli versus HFA PSD): 0.36, p<0.001 Bland-Altman analysis not performed

AUC = area under the curve, AVA = Advance Vision Analyzer, CFA = C3 fields visual field analyzer, CI = confidence interval, HFA = Humphrey Field Analyzer, ICC = Intraclass correlation coefficient, LOA = limits of agreement, MD = mean defect, MS = mean sensitivity, PSD = pattern standard deviation, r = correlation coefficient, SAP = standard automated perimetry, SD = standard deviation, SITA = Swedish Interactive Threshold Algorithm, VFI = visual field index, VRP = virtual reality perimetry, VVP = Vivid Vision Perimetry.

Name of data extractor: N.H.

Date of data extraction: October 9, 2024.

### Risk of bias assessment

The results of risk of bias assessment are shown in **Table 2**. Given that no randomized studies were included, all studies were evaluated using the Newcastle-Ottawa Scale. Risk of bias was generally moderate (64% of studies); 36% of studies had low risk of bias, and no studies had high risk of bias. Only 1 study received the maximum stars (9 stars) in all domains; only 1 study received the maximum number of stars for Selection; only 3 studies received maximum stars for Comparability; all studies received maximum stars for Outcome.

**Table 2:** Risk of bias assessment using the adapted Newcastle-Ottawa tool.

Author (Year)	Risk of bias domains			Total stars*	Risk of bias assessment
	Selection (5 stars maximum)	Comparability (1 star maximum)	Outcome (3 stars maximum)		
Stapelfeldt et al. (2021) [1]	3	0	3	6	Moderate
Grau et al. (2023) [7]	3	1	3	7	Low
Ahmed et al. (2022) [8]	4	0	3	7	Low
Wroblewski et al. (2014) [10]	3	0	3	6	Moderate
Narang et al. (2021) [11]	3	1	3	7	Low
Narang et al. (2022) [13]	4	0	3	7	Low
Razeghinejad et al. (2021) [14]	3	0	3	6	Moderate
Berneshawi et al. (2024) [16]	3	0	3	6	Moderate
Griffin et al. (2024) [15]	3	0	3	6	Moderate
Bradley et al. (2024) [17]	3	0	3	6	Moderate
Phu et al. (2024) [18]	5	1	3	9	Low
Greenfield et al. (2021) [19]	3	0	3	6	Moderate
Chia et al. (2023) [20]	3	0	3	6	Moderate
Mees et al. (2020) [21]	3	0	3	6	Moderate

*Risk of bias based on total stars: 0–4 (high), 5–6 (moderate), and 7–9 (low).

### Custom VR perimetry using the oculus quest

One study compared a custom VR perimetry system which runs on the Oculus Quest virtual reality headset to the Octopus 900 dynamic strategy and found that these two systems have comparable performances across various perimetry parameters [1]. The system followed the pattern of an Octopus 900 test with a background luminance of 10 cd/m^2^ and used a light stimuli of Goldmann size III which is projected onto the retina of the subject. The threshold strategy is not described in depth. The study included 70 eyes of 70 patients: 36 healthy and 34 glaucoma patients with early to moderate visual field loss. The study found high mean defect correlations between the custom VR perimetry and Octopus (Spearman, *ρ*≥ 0.75). Although the VR system was found to slightly underestimate visual field loss in participants with glaucoma (difference in MD of 1.4 dB), no bias was found with respect to eccentricity or participant age.

### Smartphone-based campimetry

Smartphone-based campimetry (Sb-C) utilizes a VR headset (VR One plus; Zeiss) with an application run on an iPhone 6 (SmartCampiTracker) to simulate a visual field testing environment corresponding to the Octopus G pattern and a Goldmann size III stimulus and a background luminance of 0.05 cd/m^2^ [7]. The test employs a full threshold staircase strategy using a standard 4/2 dB. One included study compared Sb-C to the Octopus 900 in normal and dynamic mode and found that the Sb-C shows promise for glaucoma screening and progression monitoring [7]. The study included 93 eyes of 93 participants: 19 control subjects, 11 pre-perimetric glaucoma subjects, 46 perimetric glaucoma subjects, and 17 ocular hypertension subjects. The study found strong mean sensitivity correlation between the two devices (r = 0.815; p<0.05). The authors note that the hill of vision could not be measured in the macula with this iteration due to the differences in background luminance. The Octopus had a background luminance of 1.27 db/m^2^, much higher than that of the Sb-C, attributable to the dark background on the iPhone, which ultimately limited the maximum sensitivity the Sb-C was able to test.

### Toronto portable perimeter

Similar to the Sb-C, the Toronto Portable Perimeter (TPP) utilizes a VR headset (VEM Medical Technologies), smartphone, and mobile application. The application presents Goldmann size III, IV, and V stimuli with background luminance of 10 cd/m^2^ [8]. The stimuli size is based on the threshold with size III used for high-threshold sensitivities and IV and V for low threshold sensitivities. The thresholding strategy is based on a previously published testing algorithm using Zippy Estimation of Sequential Threshold (ZEST) [9]. One study compared the TPP to the HFA 24–2 Swedish Interactive Threshold Algorithm (SITA) Standard test and included 150 eyes of 91 glaucoma subjects with mild (n = 114 eyes), moderate (n = 26 eyes), and advanced glaucoma (n = 10 eyes) [8]. They found good agreement on Bland-Altman analysis of mean deviation, with a mean difference (95% limits of agreement) of 0.21 dB (-4.25 to 4.67 dB); pattern standard deviation, with a mean difference (95% limits of agreement) of -0.13 dB (-3.72 to 3.47 dB); visual field index, with a mean difference (95% limits of agreement) of 0.66% (-10.94% to 12.26%); and test duration, with a mean difference (95% limits of agreement) of 0.65 seconds (-97.51 to 98.81 seconds) obtained with TPP and HFA.

### VirtualEye

The VirtualEye uses a head-mounted VR display with eye tracking (Arrington Research) connected to a computer. Each eyepiece has an organic light-emitting diode (OLED) microdisplay which allows for high luminance. Goldman III stimulus with a full threshold 4/2 strategy and the exact background luminance is not provided [10]. One study compared the VirtualEye (in two different modes–manual and visual grasp mode) to the HFA 24–2 SITA Standard test and included 59 eyes in manual mode and 40 eyes in visual grasp mode of 62 participants: 30 glaucoma subjects, 6 glaucoma suspect subjects, 9 “other” subjects, and 17 control subjects [10]. The “visual grasp” testing aspect is unique in that it uses the eye tracker to sense changes in gaze direction which is interpreted as evidence of a seen stimulus. Manual grasp is similar to conventional perimetry where the testee uses a mouse click to denote a seen stimulus. Overall, the study found that large visual field defects identified with the HFA were detected with VirtualEye and patients reported a high degree of usability. Generally, lower sensitivities were seen with the VirtualEye, particularly for stimuli with high dB.

### Advance Vision Analyzer (AVA)

The Advance Vision Analyzer (AVA) uses a head-mounted device with a liquid crystal display (LED) with an eye tracking system. The test uses a Goldmann size III stimulus with background luminance of 9.6 cd/m^2^ [11]. There are three testing strategies: Full Threshold, Elisar Standard, and Elisar Fast which use a 4/2 dB staircase procedure which is described in depth elsewhere [12]. Two studies have compared the AVA to HFA [11,13]. The first study compared the AVA to the HFA 24–2 SITA Standard test and included 160 eyes of 160 participants: 85 control subjects and 75 glaucoma subjects [11]. They found moderate correlation for several parameters between AVA and HFA: pointwise threshold sensitivity, sectoral mean sensitivity, mean deviation, pattern standard deviation, and test-retest variability (correlation coefficients r = 0.68–0.89, p<0.001). The second study compared the AVA to the HFA 10–2 SITA Standard test and included 112 eyes of 112 participants: 36 control subjects, 66 glaucoma subjects, and 10 glaucoma suspects [13]. Bland-Altman analysis showed good correlation for mean sensitivity, mean deviation, and pattern standard deviation between AVA and HFA. Both technologies accurately differentiated glaucomatous from non-glaucomatous eyes. Taken together, these two studies suggest a role for the AVA in diagnosing glaucoma and good correlation with clinical standard perimetric results.

### VisuALL

The VisuALL is a commercially available VR head-mounted device, and our search identified three studies comparing VisuALL to HFA. All studies used a Goldmann size III stimulus with a full threshold testing algorithm. The first study [14] used background luminance of 3 cd/m^2^ and the remaining two used background luminance of 1 cd/m^2^ [15,16]. The first compared the VisuALL 24 T algorithm to the HFA 24–2 SITA Standard test in 102 eyes of 51 participants: 26 with mild and moderate glaucoma (52 eyes) and 25 control participants (50 eyes) [14]. In this cohort, there was high correlation between the global mean sensitivity of the HFA and VisuALL in both control subjects (r = 0.5; p = 0.001) and glaucoma subjects (r = 0.8; p<0.001). The diagnostic accuracy for detecting glaucoma was high with area under the receiving operator characteristic curve of 0.98 for VisuALL compared to 0.93 for HFA (p = 0.06). Overall, this technology shows potential for detecting glaucoma, though additional studies are needed to determine its performance in those with advanced glaucoma. The second study compared the VisuALL (exact protocol not specified) to the HFA 24–2 SITA Standard test in 16 eyes of 9 glaucoma participants [16]. Although a small study, it demonstrated a statistically significant correlation between the average mean deviation between the two devices (r = 0.8793, p < 0.001), and Bland-Altman analysis showed good agreement for mean deviation (95% LOA -3.98, 7.35). Five of six Garway-Heath sectors showed a significant correlation between the two devices (p≤0.01). VisuALL was found to be significantly faster than HFA, with a median difference of 69.33 seconds (p<0.001). Overall, this study showed good MD correlation between VRP and SAP. The third study compared the VisuALL ST-24 protocol to the HFA 24–2 SITA Standard test in 43 eyes of 24 glaucoma participants [15]. The study found a strong positive correlation in mean deviation between the two devices (r = 0.871, p < 0.001). For all stages of glaucoma, mean differences in locus-specific sensitivity were near 0 dB, and the median differences in locus-locus differences were -0.3±1.5 dB between the two devices. Bland-Altman analysis demonstrated good correlation for pre-perimetric and mild glaucoma, but significant funneling for moderate and severe glaucoma indicating heteroscedasticity. Overall, this study showed good correlation between results from VRP and SAP, with a potential for VRP to evaluate patients in different stages of glaucoma.

### Radius

The Radius is a lightweight VR headset that uses background luminance of 10 cd/m^2^ and Goldmann III stimulus using a proprietary threshold testing strategy. A handheld Bluetooth VR controller is used for signal detection, with a Samsung tablet used to power the headset which also allows monitoring in real-time. Based on power limitations, the measurement range is limited to 15–40 dB. The test uses a RATA-Standard algorithm (a SITA growth pattern that divides all 54 test locations of the 24–2 test pattern into four groups) with a staircase procedure to specify the contrast level of each stimulus presented [17]. The one study of the Radius which met inclusion criteria compared this to the HFA 24–2 SITA Standard test in 100 eyes of 100 subjects: 50 with suspected or mild glaucoma and 50 subjects with moderate or severe glaucoma [17]. In this study, estimated sensitivities between Radius and HFA were not significantly different for right eyes (p = 0.017) but were significantly different for left eyes (p < 0.001); although the authors mention this to be an artifact and not expected in general. Though there was a strong correlation in mean deviation (r = 0.94), the Radius was found to estimate higher sensitivities in the 15–22 dB range compared to HFA. Additionally, Radius was found to be faster than HFA (298 seconds vs 341 seconds, respectively). Masked glaucoma experts graded glaucoma severity using results from both tests and the concordance in glaucoma staging was high (kappa = 0.91–0.93). Overall, this study showed comparable estimated sensitivities between Radius and HFA and good concordance for glaucoma severity grading.

### Virtual field using the Oculus Go

Virtual Field is a VR perimetry software that is FDA-approved for use as an automated perimeter. A Fast Full Threshold algorithm is used with a Goldmann size III stimulus with a background luminance of 0.218 cd/m^2^ [18]. One study met inclusion criteria and compared the Virtual Field using the Oculus Go virtual reality headset to the HFA 24–2 SITA Standard test in 95 eyes of 95 participants (41 control, 54 glaucoma) [18]. The study found a strong correlation in mean deviation (r = 0.87, p<0.001) and pattern standard deviation (r = 0.94, p<0.001) and a moderate correlation in pointwise sensitivity (r = 0.78, p<0.001) between the two devices. On Bland-Altman analysis, mean difference of 0.36 dB and 0.11 dB for MD and PSD, respectively (95% LOA -3.48, 4.20, p = 0.9125; 95% LOA -3.19, 3.41, p<0.0001, respectively). Compared to VRP, reliability with SAP was worse with higher fixation losses (0.13 vs 0.05, respectively; p = 0.0006) and higher false-positive rates (2% vs 1%, respectively; p < 0.0001). Virtual Field testing was significantly faster than HFA, with an average difference of 76 seconds (p<0.0001). Overall, this study showed good correlation for mean deviation and pattern standard deviation between VRP and SAP. In this cohort, Virtual Field testing was quicker and more reliable.

### Vivid Vision Perimetry (VVP; suprathreshold)

Two studies evaluated Vivid Vision Perimetry (VVP), which is software that can be installed onto off-the-shelf VR headsets. The studies of this device used an Oculus Go mobile virtual reality headset (Facebook, Inc). The test uses a black Goldmann size III stimulus with a white background with luminance of 25 cd/m^2^. This suprathreshold test presents stimuli in random locations. If the first stimulus in a location is seen, it is not presented again. If missed, it is presented 2 additional times [19]. One study compared the VVP Swift to the HFA 24–2 SITA Standard test and included 24 eyes of 12 participants: 7 with glaucoma and 5 with glaucoma suspect [19]. This was a small study but demonstrated good reproducibility and a statistically significant association between VVP Swift average mean sensitivity and HVF mean deviation (r = 0.86; p<0.001). The second study evaluated the VVP-10 test to the HFA 24–2 SITA Standard test and included 36 eyes of 21 participants with mild, moderate, and advanced glaucoma [20]. For all glaucoma eyes, the correlation between VVP average fraction seen compared to HVF mean sensitivity was 0.67 (p<0.05). Overall, these studies demonstrate that VVP in its current iteration has moderate to strong correlation with HFA testing, but additional data is needed to understand if this technology is suited to detect mild glaucoma.

### C3 fields visual field analyzer (CFA; suprathreshold)

The C3 fields visual field analyzer (CFA) is a head-mounted VR perimeter which uses a suprathreshold test with a 0.55 mm stimulus with 60 cd/m^2^ luminance and background luminance of 4 cd/m^2^. The test follows the pattern of the 24–2 SITA algorithm and a stimulus is shown twice in each location [21]. One study compared the CFA to the HFA 24–2 SITA Standard test in 157 participants: 62 with mild, moderate, and advanced glaucoma and 95 control participants [21]. In this study, the CFA did not reliability identify deficits identified with HFA. It was moderately effective at identifying glaucoma subjects and performed better in identifying glaucoma in participants with moderate and advanced disease compared to those with mild disease (area under the receiving operator characteristic curve of 0.77 for mild glaucoma and 0.86 for moderate to advanced glaucoma).

### Prior experience with SAP testing

There was some variability and incomplete data in reporting participant exposure to prior visual field testing, with only eight of the included studies reporting information on this. For Sb-C, VirtualEye, VVP Swift, one of the VisuALL studies, and Radius, all recruited participants had prior experience with HFA [7,10,15,17,19]. For TPP and Virtual Field, the authors noted that most patients had prior experience with HFA testing [8,18]. For AVA, all patients with glaucoma had prior experience with perimetry, and this data was not reported for participants without glaucoma [11].

### User experience with VR perimetry

Overall, four of the included studies specifically reported on user experience using VRP. For TPP, the authors administered a 5-question survey with results calculated as proportions to assess test preference, comfort, ease, and testing location preference [8]. Participants preferred the experience of testing with the TPP (P<0.001), reported that the TPP had easier to understand test instructions (P<0.001), and noted that testing was easier to perform with the TPP compared to the HFA (P = 0.007). No difference was found regarding anxiety levels during testing between the two, but participants preferred completing testing at home compared to in clinic (P<0.001). For the VirtualEye, the authors administered a 6-question survey graded on a scale of 1 to 5 to assess comfort, difficulty, and device preference. Participants preferred the experience of testing with VirtualEye compared to HFA (median rating of 2 with 1 representing VirtualEye and 5 representing HFA). This preference reflected high scores for both comfort and ease of use of the VirtualEye device [10]. No difference was found between the two testing modes (manual versus visual grasp). For the VVP-10, the authors evaluated patients’ discomfort and fatigue using a 5-point Likert scale, with 1 = no discomfort or fatigue, and 5 = extremely high discomfort or fatigue [20]. Participants reported similar discomfort (P = 0.51) and fatigue (P = 0.09) levels between VVP-10 and HFA testing, with average Likert scores near 2/5. Lastly, for CFA, a similar 6-question survey was administered to evaluate satisfaction with the CFA compared to the HFA [21]. Results were converted to a scale of 1 to 5. Detailed results of the survey are not included, but participants reported that the CFA was more comfortable and easier to use (P<0.001) and on forced choice, 93% preferred the CFA.

## Discussion

Historically, SAP has been the standard of care for visual field testing in glaucoma. Among other limitations, the subjective nature, time and positioning requirements have revealed the need for innovative methods to perform visual field testing. This systematic review explored the available data of existing VRP systems compared to SAP in adults with glaucoma. Overall, there was variability by device, but in general, early data is promising. However, more work is required to better evaluate these devices. Specifically, around half of the studies lacked rigorous Bland-Altman analysis, and data regarding patient acceptability and usability was sparse. Additionally, most studies did contain heterogeneous populations, which could introduce spectrum bias.

In general, VRP seemed to underestimate MS and defect size for glaucoma patients and overestimate for healthy patients, except for TPP, which overestimated relative to HFA [7,8,10,11,13,14,19–21]. In addition, while most VRPs studied reliably produced results comparable to those of the clinical standard, CFA failed to yield similar results [21]. However, given the statistical similarity of VRP MS and MD to that of HFA, the literature provides some evidence that VRP may be used to distinguish between glaucomatous and healthy eyes and assess visual fields [1,7,8,10,11,13,14,19–21]. Despite the strong association between VRP parameters and those of the clinical standard, there are questions that remain: 1) does the reliability of testing vary significantly with severity of disease? 2) how sensitive is VRP to changes associated with progression of glaucomatous disease? 3) does VRP have any utility in glaucoma patients with comorbid ocular conditions? There are also technical limitations to VRP including heterogeneity in eye tracking capability and luminance.

Limitations of the studies included both those inherent to the testing modality as well as those evident in the study design and participants. Investigators from several studies noted that patients with mild glaucoma may be overrepresented relative to those with moderate and advanced forms, which can lead to an overestimation of diagnostic accuracy due to spectrum bias [8,14,22]. This is clinically relevant because the literature is suggestive of the idea that the performance of VRP may vary with the severity of glaucoma [1,19–21]. Greenfield et al. noted an inverse relationship between glaucoma severity and the precision of VVP, while Stapelfeldt et al. noticed an inverse relationship between glaucoma severity and detection of defects for Oculus Quest [1,19]. In addition, although Narang et al. noted no significant difference between MS and MD results obtained from AVA and HFA for glaucoma, this determination was made using global analyses rather than pointwise analyses, which may not be sensitive enough to detect small defects [13]. This idea was also demonstrated by Chia et al. and Mees et al., who noted an inverse relationship between the correlation of VVP and CFA parameters to HFA parameters when less advanced disease/smaller defects were present [20,21]. From this, it follows that if VRP cannot reliably detect early glaucomatous damage, recommendations for annual frequency of testing with VRP will differ by device and deviate from that of the clinical standard.

Additionally, several studies noted the demographic differences between participant groups as a potential source of bias. In the study by Chia et al., the lack of racial diversity and the small sample size may limit the generalizability of the results [20]. Authors also noted the lower average age of their participants as a potentially contributing factor, given the possibility that younger patients are more likely to be familiar with and receptive to virtual reality technology [14,21]. Finally, nonrandomized order of testing created the potential for skewing of results in several studies [7,10,14,16,19,21]. In the setting of nonrandomized testing order, learning bias and testing fatigue can influence the results of testing. Furthermore, the lack of standardization of testing parameters limits the direct comparison of these results. Lastly, in terms of the VRP devices themselves, while SAP accurately measures distance, the simulated distance emulated by VRP devices may not accurately correspond with the true distances used in SAP. To establish VRP as a reliable tool for glaucoma diagnosis and monitoring, future research should focus on developing transparent standardized testing protocols. Additionally, providing publicly-available diverse normative databases, test-retest reliability data, and clear guidelines for interpreting results across perimetry modalities would be useful. Finally, all studies assessing the diagnostic accuracy of novel VRP devices should follow Standards for Reporting of Diagnostic Accuracy Studies (STARD) guidelines [23].

Limitations of the current study include studies that may have been missed from other databases; however, we hope to have captured all relevant studies with our database search. Also, our pre-specified plan did not include performing a meta-analysis, but this may be considered as additional studies arise. Additionally, although the search and screening strategy was robust, new VR technologies and software iterations continue to emerge and may have been missed.

In conclusion, our systematic review identified 14 studies comparing 10 different virtual reality perimetry systems with standard automated perimetry in adults with glaucoma. These studies add to the growing body of evidence on the utility and feasibility of virtual reality perimetry, which can help providers treat and monitor certain at-risk glaucoma patients. Further research is necessary to evaluate the utility of VRP within the context of both advanced and early glaucoma and its applicability beyond screening. Subsequent investigations should include larger, more representative sample sizes, pointwise comparisons, and randomized testing orders.

## Supporting information

S1 Checklist PRISMA 2020 checklist. (DOCX)

S1 Protocol Protocol with predefined search strategy used for the systematic review. (DOCX)

S1 Appendix Search strategy for pubmed central, embase, and cochrane central register of controlled trials databases. (DOCX)

S1 Table Studies identified in the literature search. (XLSX)
