# Supplementary material for: Virtual reality perimetry compared to standard automated perimetry in adults with glaucoma: A systematic review
Source: PLoS One. 2025 Jan 24;20(1):e0318074. doi: 10.1371/journal.pone.0318074 (PMC11760034; doi:10.1371/journal.pone.0318074)
Supplement: S1 Protocol — (DOCX) [file pone.0318074.s002.docx]

**Supplemental Protocol 1.** Protocol with predefined search strategy used for the systematic review

**TITLE:** Virtual Reality Perimetry in Glaucoma: A Systematic Review

**MOTIVATION**

Glaucoma is one of the leading causes of blindness and affects 80 million people worldwide.^1^Standard automated perimetry (SAP) using the Humphrey Field Analyzer (HFA) or Octopus is generally considered the gold standard test to monitor disease progression, however test duration and patient concentration can often limit the quality of results (and willingness to participate in testing). Newer technologies are necessary to provide accessible and innovative ways of assessing visual field loss.^2^

Virtual reality perimetry (VRP), using virtual reality headsets to perform visual field testing, has gained increasing attention due to its portability and potentially improved acceptability among patients.^3^ While VRP has been shown to detect visual field defects with some correlation to the gold standard HFA, there has been no systematic review evaluating the evidence for VRP in glaucoma. This study aims to consolidate available data in a systematic review of the utility of VRP in management of glaucoma using mean defect (MD) analysis.

**REVIEW QUESTION**

To assess the utility of VRP as an alternative to HFA in visual field testing for glaucoma

**SEARCH STRATEGY**

- Search terms
  - Virtual reality
  - Olleyes
  - Vivid Vision
  - VirtualEye
  - Advanced Vision Analyzer (AVA)
  - Kasha Visual Field System
  - Virtual Field
  - Glaucoma
- Synonyms to be searched
  - AVA
- Search algorithm
  - (virtual reality OR Olleyes OR Vivid Vision OR VirtualEye OR Advanced Vision Analyzer OR Kasha Visual Field System OR Virtual Field) AND (glaucoma)
- Databases to search
  - Pubmed
  - EMBASE
  - Cochrane Library
- Other sources
  - References of relevant review literature identified through the above searches will be hand-reviewed to identify relevant studies

**Study Selection Process**

- Querying above databases and additional sources to gather all relevant articles
- Removing duplicates
- Screening based on inclusion and exclusion criteria by one person
- Full text review and extraction of relevant data into Excel by one person

**Article Eligibility Criteria**

- **Inclusion criteria**
- Study design: randomized controlled trial, prospective cohort study, or retrospective cohort study
- Contains patients undergoing both SAP and some form of VRP
- Device types:
  - Olleyes
  - Vivid Vision
  - VirtualEye
  - Advanced Vision Analyzer (AVA)
  - Kasha Visual Field System
  - Oculus Quest
  - Sb-C
  - C3 Field Analyzer
- Studies for which the primary outcome includes some quantitative correlation comparing SAP and VRP results
- **Exclusion criteria**
  - Unrelated/irrelevant
  - Reviews articles, letters, case reports, abstract-only papers, unavailable full text
  - Non-English language papers

**OUTCOMES**

- **Primary outcome of interest**
  - Mean deviation (MD)
  - Mean sensitivity (MS)
  - Degree of correlation
- **Secondary outcomes**
  - Test duration between devices
  - Test performance based on severity of visual field loss (as assessed by SAP MD)
  - Results of sectoral or pointwise comparison
  - Ease of use for VRP

**DATA EXTRACTION**

- Variables to extract
  - Year of publication of study
  - Location of study (state and country)
  - Study setting (in office or home testing)
  - Study design
    - 1 = RCT
    - 2 = prospective cohort study
    - 3 = retrospective cohort study
    - 4 = other
  - VRP Studied
    - 1 = Olleyes
    - 2 = Vivid Vision
    - 3 = VirtualEye
    - 4 = AVA
    - 5 = Kasha
    - 6 = Oculus Quest
    - 7 = Sb-C
    - 8 = C3 Field Analyzer
  - Sample size (number of eyes, number of patients)
    - Sample size of total group
    - Sample size of each subgroup, if any
  - Demographic information
    - Mean, standard deviation, median, range Age at time of testing
    - Sex
  - Primary outcome data:
    - - MD difference and SD
      - MS difference and SD
      - Correlation coefficient
  - Secondary outcome data:
    - Test duration between devices
    - Sub-group analysis based on severity of visual field loss if available
    - Results of sectoral or pointwise comparison
    - Ease of use for VRP

**Analyses**

- **Strategy for data synthesis (qualitative, quantitative)**
  - A qualitative synthesis of individual studies will be performed to discuss individual findings per the variables extracted.
- **Subgroups**
  - Different VRP devices
  - Standard SAP (HFA or Octopus)
  - Correlation variation based on severity of visual field loss
- **Quality assessment**
  - Risk of bias will be assessed using the Cochrane risk of bias tool for randomized controlled trials and the Newcastle-Ottawa Scale for non-randomized studies

**Registration Citation**

- PROSPERO: CRD42023429071
- Date of Registration: May 23, 2023
- Available from: https://www.crd.york.ac.uk/PROSPERO

**References**

1. Stapelfeldt J, Kucur SS, Huber N, Höhn R, Sznitman R. Virtual Reality-Based and Conventional Visual Field Examination Comparison in Healthy and Glaucoma Patients. *Transl Vis Sci Technol*. 2021;10(12):10. doi:10.1167/tvst.10.12.10
2. Glen FC, Baker H, Crabb DP. A qualitative investigation into patients' views on visual field testing for glaucoma monitoring. *BMJ Open*. 2014;4(1):e003996. Published 2014 Jan 10. doi:10.1136/bmjopen-2013-003996
3. Hu GY, Prasad J, Chen DK, Alcantara-Castillo JC, Patel VN, Al-Aswad LA. Home Monitoring of Glaucoma Using a Home Tonometer and a Novel Virtual Reality Visual Field Device: Acceptability and Feasibility. *Ophthalmology Glaucoma*. Published online May 14, 2022:S2589-4196(22)000813. Doi: https://doi.org/10.1016/j.ogla.2022.05.001
