# Supplementary material for: Virtual reality perimetry compared to standard automated perimetry in adults with glaucoma: A systematic review
Source: PLoS One. 2025 Jan 24;20(1):e0318074. doi: 10.1371/journal.pone.0318074 (PMC11760034; doi:10.1371/journal.pone.0318074)
Supplement: S1 Appendix — (DOCX) [file pone.0318074.s003.docx]

**Appendix** — Search Strategy for PubMed Central, Embase, and Cochrane Central Register of Controlled Trials databases

Database: PubMed Central

1. Visit webpage at <https://www.ncbi.nlm.nih.gov/pmc/>
2. In the “Search in PMC” search box, each of the following terms were searched in the following order:
   1. "Virtual Reality" AND glaucoma
   2. (("Glaucoma"[Majr])) AND "Virtual Reality"[Mesh:NoExp]
   3. "Olleyes" AND glaucoma
   4. "Vivid Vision" AND glaucoma
   5. “VirtualEye” AND glaucoma
   6. "AVA" OR "Advanced Vision Analyzer" AND glaucoma
   7. "Kasha Visual Field System" AND glaucoma
3. The number of PubMed Central Full-Text Search Results were recorded into an Excel spreadsheet

Database: Embase

1. Visit webpage at <https://www.embase.com/search/quick>
2. In the “Broad search” search box, each of the following terms were searched in the following order:
   1. "Virtual Reality" AND glaucoma
   2. (("Glaucoma"[Majr])) AND "Virtual Reality"[Mesh:NoExp]
   3. "Olleyes" AND glaucoma
   4. "Vivid Vision" AND glaucoma
   5. “VirtualEye” AND glaucoma
   6. "AVA" OR "Advanced Vision Analyzer" AND glaucoma
   7. "Kasha Visual Field System" AND glaucoma
3. The number of search results were recorded into an Excel spreadsheet

Database: Cochrane Central Register of Controlled Trials

1. Visit webpage at <https://www.cochranelibrary.com/central/about-central>
2. By the search box, “All Text” was selected
3. In the search box, each of the following terms were searched in the following order:
   1. "Virtual Reality" AND glaucoma
   2. (("Glaucoma"[Majr])) AND "Virtual Reality"[Mesh:NoExp]
   3. "Olleyes" AND glaucoma
   4. "Vivid Vision" AND glaucoma
   5. “VirtualEye” AND glaucoma
   6. "AVA" OR "Advanced Vision Analyzer" AND glaucoma
   7. "Kasha Visual Field System" AND glaucoma
4. The number of trials matching were recorded into an Excel spreadsheet
